# Synthesis, In Vitro Biological Evaluation and In Silico Molecular Docking Studies of Indole Based Thiadiazole Derivatives as Dual Inhibitor of Acetylcholinesterase and Butyrylchloinesterase

**DOI:** 10.3390/molecules27217368

**Published:** 2022-10-29

**Authors:** Shoaib Khan, Shahid Iqbal, Muhammad Taha, Fazal Rahim, Mazloom Shah, Hayat Ullah, Ali Bahadur, Hamad Alrbyawi, Ayed A. Dera, Mohammed Issa Alahmdi, Rami Adel Pashameah, Eman Alzahrani, Abd-ElAziem Farouk

**Affiliations:** 1 Department of Chemistry, Hazara University, Mansehra 21120, Pakistan; 2 Department of Chemistry, School of Natural Sciences (SNS), National University of Science and Technology (NUST), H-12, Islamabad 46000, Pakistan; 3 Department of Clinical Pharmacy, Institute for Research and Medical Consultations (IRMC), Imam Abdulrah-Man Bin Faisal University, Dammam 34212, Saudi Arabia; 4 Department of Chemistry, Abbottabad University of Science and Technology (AUST), Abbottabad 22500, Pakistan; 5 Department of Chemistry, University of Okara, Okara 56300, Pakistan; 6 Department of Chemistry, College of Science and Technology, Wenzhou-Kean University, Wenzhou 325060, China; 7 Pharmaceutics and Pharmaceutical Technology Department, College of Pharmacy, Taibah University, Medina 42353, Saudi Arabia; 8 Department of Clinical Laboratory Sciences, College of Applied Medical Sciences, King Khalid University, Abha 61413, Saudi Arabia; 9 Department of Chemistry, Faculty of Science, University of Tabuk, Tabuk 71491, Saudi Arabia; 10 Department of Chemistry, Faculty of Applied Science, Umm Al-Qura University, Makkah 24230, Saudi Arabia; 11 Department of Chemistry, College of Science, Taif University, Taif 21944, Saudi Arabia; 12 Department of Biotechnology, College of Science, Taif University, Taif 21944, Saudi Arabia

**Keywords:** indole, thiadiazole analogs, SAR, AchE and BuChE inhibitors, molecular docking

## Abstract

The current study was conducted to obtain hybrid analogues of indole-based thiadiazole derivatives (**1**–**16**) in which a number of reaction steps were involved. To examine their biological activity in the presence of the reference drug Donepezil (0.21 ± 0.12 and 0.30 ± 0.32 M, respectively), the inhibitory potentials of AChE and BuChE were determined for these compounds. Different substituted derivatives showing a varied range of inhibitory profiles, when compared to the reference drug, analogue **8** was shown to have potent activity, with IC50 values for AchE 0.15 ± 0.050 M and BuChE 0.20 ± 0.10, respectively, while other substituted compounds displayed good to moderate potentials. Varied spectroscopic techniques including ^1^H, ^13^CNMR and HREI-MS were used to identify the basic skeleton of these compounds. Furthermore, all analogues have a known structure–activity relationship (SAR), and molecular docking investigations have verified the binding interactions of molecule to the active site of enzymes.

## 1. Introduction

Alzheimer’s disease is associated with the acetylcholinesterase (AChE) and butyrylcholinesterase (BuChE) enzymes, which damage the human brain. The conversion of acetylcholine into choline acetic acid by the process of hydrolysis is the primary function of these enzymes [1]. Owing to these consequences, the hippocampus and cortex of the brain, which are linked to important psychological functions, suffer from acetylcholine deficiency [2]. As a result of this ongoing process, the patient develops Alzheimer’s disease (AD), an irreversible brain illness. In addition, the brain’s continually disrupted cholinergic system frequently results in cognitive impairment, difficulties thinking, confusion, difficulty solving problems, and memory loss [3,4,5]. Additionally, due to the enzymes that generate neurotoxic beta amyloid accumulation, which results in neuronal cell death, AD is regarded as the primary cause of dementia in an ageing society. The primary goal of targeting both of these enzymes is to prevent and cure AD [6,7]. It induces neurotoxicity as a consequence of its interaction with protein by specifically targeting the acetylcholinesterase enzyme, which has two binding sites, including a peripheral site for beta-amyloid association and a catalytic site for acetylcholine hydrolysis.

Furthermore, cholinergic neurons, muscles, and the brain all contain acetylcholinesterase, whereas the liver, heart, kidneys, lungs, heart, and intestines are the primary locations of butyrylcholinesterase [8,9]. As cholinergic activity steadily decreases, the main function of these enzymes is to breakdown the ester-containing analogues, among which AChE is dominant in the brain. Therefore, a significant drug is still required to reduce these enzymes’ potential [10]. The FDA has licensed a number of drugs, including rivastigmine, Donepezil, galantamine, and tacrine for the treatment of Alzheimer’s disease [11]. Moreover, due to the drug’s gastrointestinal upset, inadequate action, and hepatotoxicity, its use and application are limited [12,13,14,15,16]. We have synthesized hybrid indole-based thiadiazole derivatives as a novel class of effective inhibitors in response to the present situation of Alzheimer’s disease and the absence of effective moieties. Among the heterocyclic compounds, indole is the most important and is mostly found in the secondary metabolites of many natural products, including alkaloids, the secondary metabolites of fungi, and the secondary metabolites of marine organisms, among others [17]. Indole moiety is a key component of medicinal chemistry and is employed as a precursor in a variety of chemical syntheses. The indole nucleus has a wide range of biological potentials because of its extensive biological profile including anticancer, anti-ulcerative, antimalarial, anti-platelet, anti-leishmanial, anti-oxidant, anti-rheumatoid, antibacterial, anti-HIV [18,19,20,21,22,23,24,25,26], Leukotriene B4 tyrosinase, aldose (reductase potential), immune-modulators, and substances that prevent the release of chemical mediators are anti-tubercular [27,28]. This research seeks to look into promising indole-based thiadiazole compounds. Among the synthesized series, all analogs showed moderate to good inhibitory potentials except analog 8, which was found as the greatest inhibitor of both targeted enzymes with minimum IC_50_ values of 0.15 0.050 µM (for AChE) and 0.20 ± 0.10 µM (for BuChE).

## 2. Result and Discussion

### 2.1. Chemistry

**Scheme 1:**
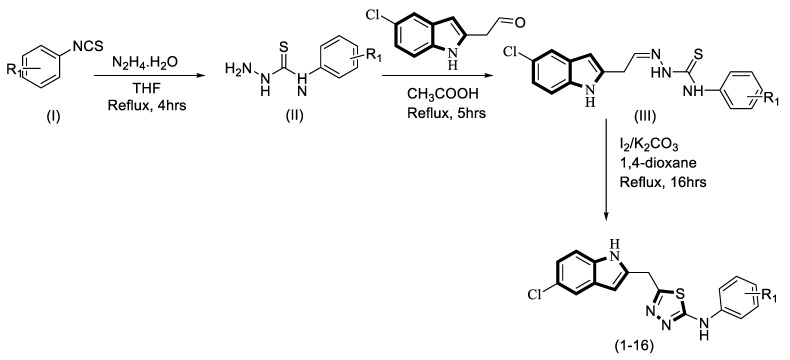
Representation of the stepwise (I-III) procedure adopted for the synthesis of indole-based thiadiazole moieties (**1–16**).

In order to synthesize thiosemicarbazide derivatives (III), various substituted isothiocyanates and hydrazine hydrate were combined in THF and refluxed the reaction mixture for approximately 4 h. These derivatives were then treated with an indole molecule containing an aldehyde moiety in the presence of acetic acid in the refluxing conditions for about 5 h, yielding Schiff bases as an intermediate (III). These intermediates were cyclized after 16 h refluxed in 1,4-dioxane in the presence of I_2_/K_2_CO_3_, and we obtained (Table 1) indole based thiadiazole derivatives (**1**–**16**). The stepwise reaction procedure is shown in Scheme 1.

### 2.2. Spectral Analysis

Spectral analyses of all the synthesized compounds have been discussed, and their respective spectra’s have been added in Supplementary Information as shown in Figures S1–S3.

Different substitution patterns of the compounds play a vital role to shield or de-shield the proton and carbon atoms. All the synthesized compounds were found with different yields in the final products and confirmed through thin layer chromatography (TLC). After TLC confirmation, the compounds were washed with *n*-hexane to obtained pure products, which were then characterized through ^1^H-NMR, 13C-NMR and HREI-MS techniques. The basic skeleton of the analogues were obtained and confirmed the nature of functionality attached to the aromatic ring. Spectral interpretation was done and their values were written in descending order for both proton and carbon (as shown in Supplementary Information). Furthermore, mass spectroscopic techniques confirmed the molecular weight of the synthesized compounds. Structure interpretation for compound-9 in which proton appeared at varied ranges (ppm). Using ^1^HNMR (600 MHz, DMSO-*d_6_*): *δ*, the first proton appeared at 11.59, showing a singlet by one proton of nitrogen and 11.20 another proton of nitrogen appeared. The indole proton appeared at 8.15 ppm showing a doublet with a coupling constant 1.8 Hz (meta-coupling), 8.84 showing a doublet of doublet with a coupling constant 7.5 and 2.3 Hz for an aromatic proton, 7.78 showing a doublet of doublet with a coupling constant 7.1, 1.9 Hz for an aromatic proton. Multiple were shown in the range between 7.65–7.64 and 7.45–7.41 by two aromatic protons, 7.20 showing a doublet for the indole proton with a coupling constant 8.3 Hz, while the other indole proton also appeared at 6.45 showing a doublet with a coupling constant 6.7 Hz and 6.47 showing a singlet for one proton. Additionally, the CH2 proton appeared at 3.20 showing a singlet for two protons. Carbon NMR also represents the following values appearing in different ranges due to the attached substituent. Using ^13^C-NMR (150 MHz, DMSO-d*_6_*) *δ*, the carbons appeared in the range from 150.6–45.6 and the High Resolution Electron Impact Mass Spectroscopy were conducted to figure out m/z 358.0274; [M+1]^+^ Calcd for C_17_H_12_ClFN_4_S;358.0292.

### 2.3. An Illustration of a Molecule

**Figure 1:**
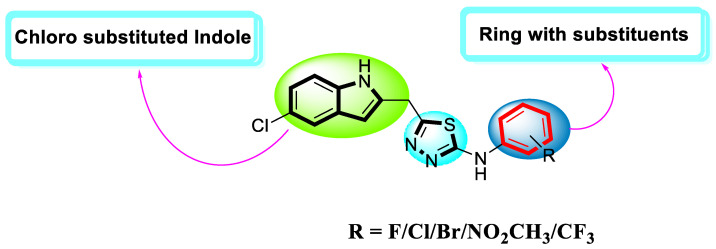
Representation of different parts of a molecule as an effective inhibitor of AchE and BuChE.

The comparison of identically substituted derivatives was carried out using the general structure, which divides the molecule into two distinct components, such as the chloro-indole moiety and aromatic ring with various substituents (Figure 1). These different parts of molecules play a key role in the inhibitory activity of the analogues and might be the presence of attached substituents upon it. The inhibitory profile depends on nature, number and position of the substituents.

## 3. Bio-Activity of Indole Based Thiadiazole Derivatives

### Inhibitory Profile of Synthesized Compound against AchE and BuChE

The synthetic scaffolds’ AchE and BuChE inhibitory profiles were evaluated in the presence of the reference medication Donepezil. Due to the presence of the electron-donating group, the majority of the compounds were discovered to have higher potential.

These scaffolds were examined along with their capacity to inhibit acetylcholinesterase and butyrylcholinesterase (**1–16**). The inhibitory potency of many substituted derivatives varied when compared to the reference drug Donepezil (IC_50_ = 0.21 ± 0.12 and 0.30 ± 0.32 μM, respectively), and several substituted derivatives displayed varying inhibitory capability, with IC_50_ values ranging from 0.15 ± 0.050 to 32.10 ±0.60 and 0.20 ± 0.10 to 37.30 ± 0.60 μM, respectively. When the same substituent was present on an aromatic ring at various positions, AChE and BuChE inhibitions were performed in order to test these analogues and compare their effects. By comparing analogues 1–3 with varying positions for the nitro group on the aromatic ring, fluctuations were observed in the inhibitory profile against AChE and BuChE. Analog **1** (2.10 ± 0.10 and 2.80 ± 0.20 μM), **2** (1.90 ± 0.10 and 4.30 ± 0.10 μM), and **3** (2.90 ± 0.10 and 4.60 ± 0.10 μM), respectively. It was determined that analogue 2 showed substantial activity, which may be caused by the presence of a chloro-group in the aromatic ring’s ortho-position, whereas analogues 1 and 3 with chloro-groups in their respective para- and meta-positions had considerably lower activity profiles than analogue 2.

Moreover, other substituted analogues such as compounds having a methyl group on varied positions of the ring containing analog **5** (5.80 ± 0.20 and 9.30 ± 0.10 μM) **6** (8.70 ± 0.20 and 10.90 ± 0.10 μM), and **7** (9.60 ± 0.20 and 13.60 ± 0.30 μM), are composed of two methyl groups in different positions, such as para, ortho, and meta. The para > ortho > meta substituted scaffold was confirmed by the IC50 values of the enzyme inhibition pattern. It was readily recognized that the methyl group’s orientation on the aromatic ring caused a difference in the inhibition activity. Analog 8 was a candidate in this comparative study of methyl-substituted compounds, however due to the location of the substituent on the aromatic ring, it demonstrated insufficient activity. The flouro-group-containing scaffolds **8** (0.15 ± 0.050 and 0.20 ± 0.10 μM), 9 (0.35 ± 0.050 and 0.50 ± 0.050 μM), and 10 (1.10 ± 0.10 and 2.80 ± 0.10 μM) displayed varying levels of inhibition against AChE and BuChE. The inhibitory potential of the compound was shown to be increased by the flouro-group in the para-position. Due to the strong hydrogen bond interaction with the enzyme active site, analogue 8 was thus regarded as the most active molecule in the tested series and demonstrated high activity when compared to the standard drug. Due to the presence of the flouro-group in the ortho-position, analogue **9** got the second highest ranking in the inhibitory profile. Methoxy substituted analogues **12** (14.70 ± 0.30 and 19.20 ± 0.30 μM) and **13** (19.10 ± 0.30 and 25.30 ± 0.40 μM) bearing nitro- and methoxy-groups at different position of aromatic ring -OMe substituted analogues **12** (14.70 ± 0.30 and 19.20 ± 0.30 μM) and **13** (19.10 ± 0.30 and 25.30 ± 0.40 μM), bearing –NO_2_ and -OMe groups at different position of aromatic ring were contrasted in order to investigate their efficacy to inhibit AChE and BuChE, respectively. Comparing these analogues to the reference drug Donepezil, they demonstrated moderate to poor inhibitions (IC50 = 0.21 ± 0.12 and 0.30 ± 0.32 μM, respectively).

The AChE and BuChE inhibitory action may slightly increase or decrease depending on the type, number, and orientation of the substituents. It was discovered that analogues **8** and **9** were slightly more capable than the reference drug Donepezil. The better interactions of the molecule that were found might be due to the attached functionality of the compounds, which make hydrogen bond in order to inhibit the negative effect of enzymes; thus, the reduction of enzymatic activity was observed and confirmed the potency of the molecules. In this study, compound **8** and **9** were found with very significant characters and considered as potent analogs when we compared their inhibitory profile with the standard drug Donepezil.

## 4. Molecular Docking

The primary linkage between ligands and the targeted enzymes has been identified using molecular docking studies. Several software, such Auto Dock Vina and Discovery Studio Visualizer, were used. [29,30,31,32,33,34]. Proteins were retrieved for this work by searching for their codes, such as 1Acl and 1p0p, in the online protein data bank (RCSB PDB).

**Figure 2:**
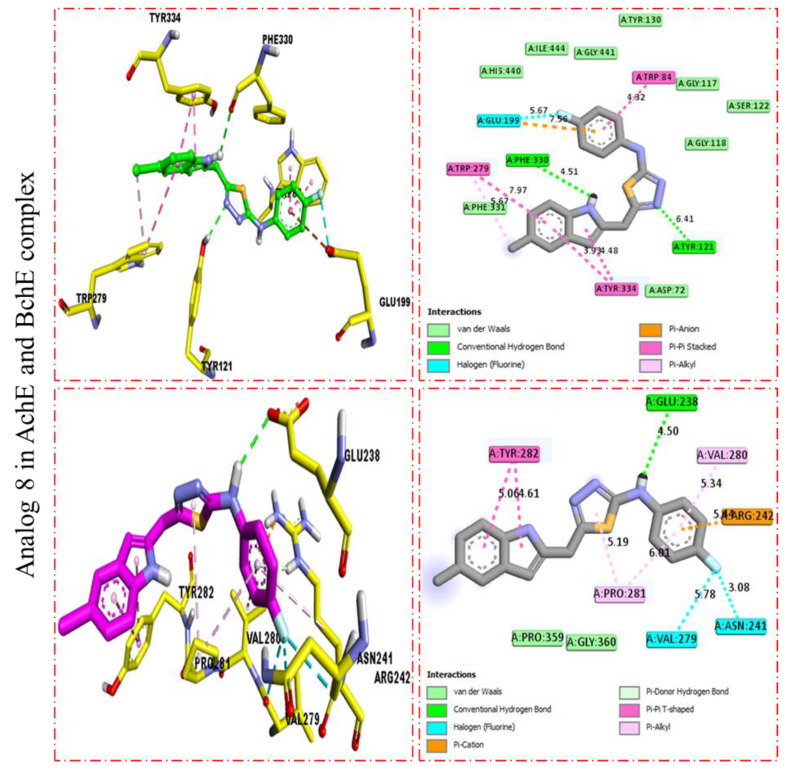
Representation of varied interaction of ligand with enzyme active sites of compound **8** against AchE and BuchE.

**Figure 3:**
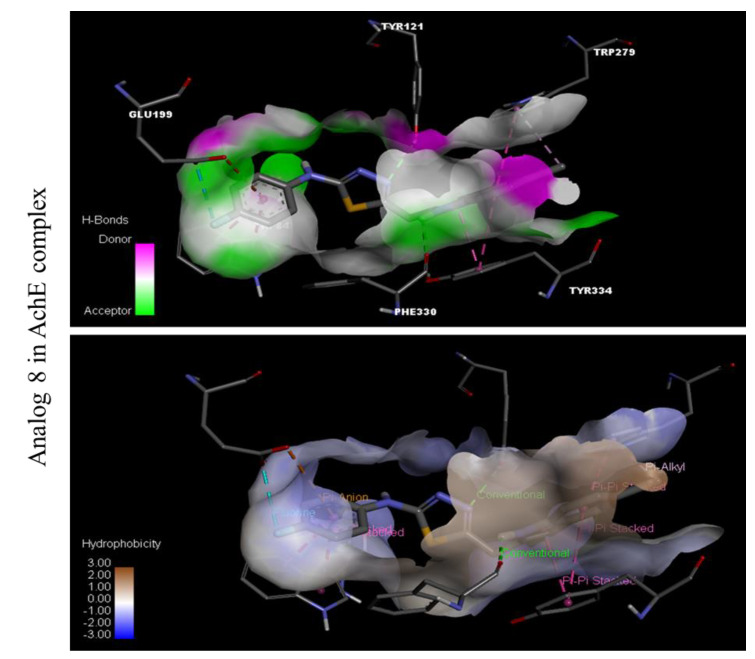
Indication of the hydrogen bond and hydrophobic nature against AChE of compound **8**.

**Figure 4:**
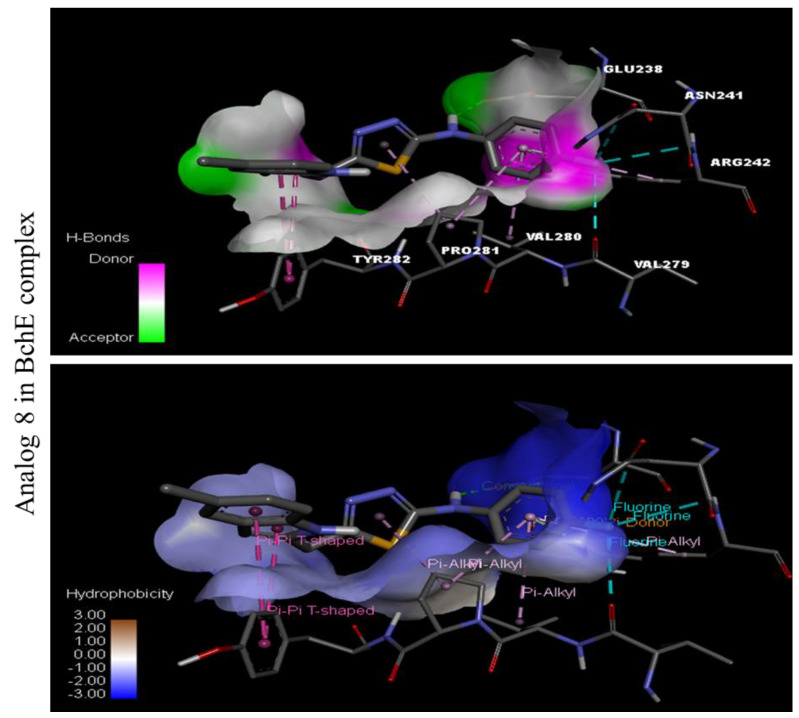
Indication of the hydrogen bond and hydrophobic nature against BuChE of compound **8**.

**Figure 5:**
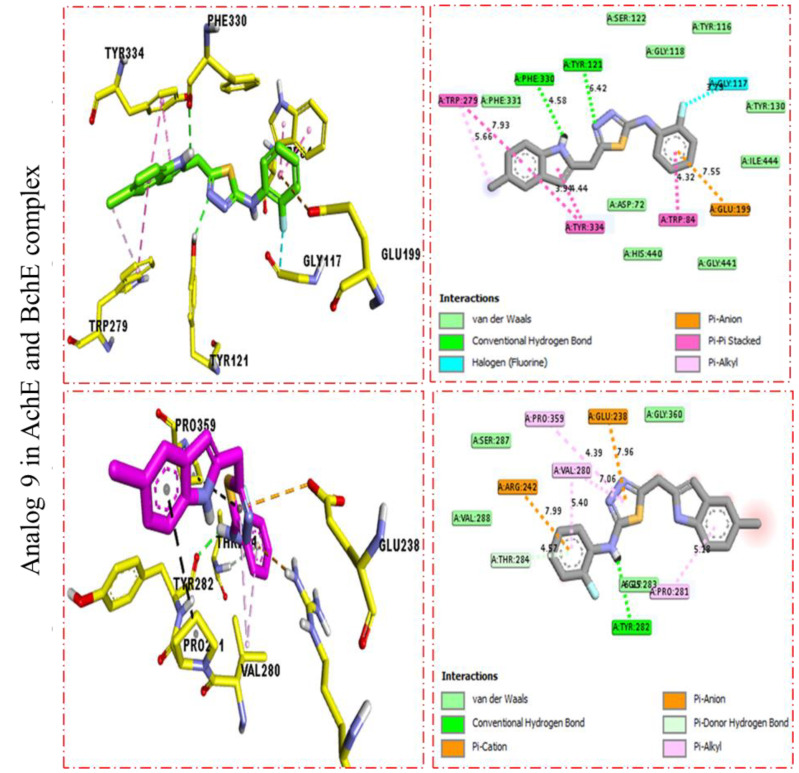
Representation of varied interaction of ligand with enzyme active sites of compound **9** against AchE and BuchE.

**Figure 6:**
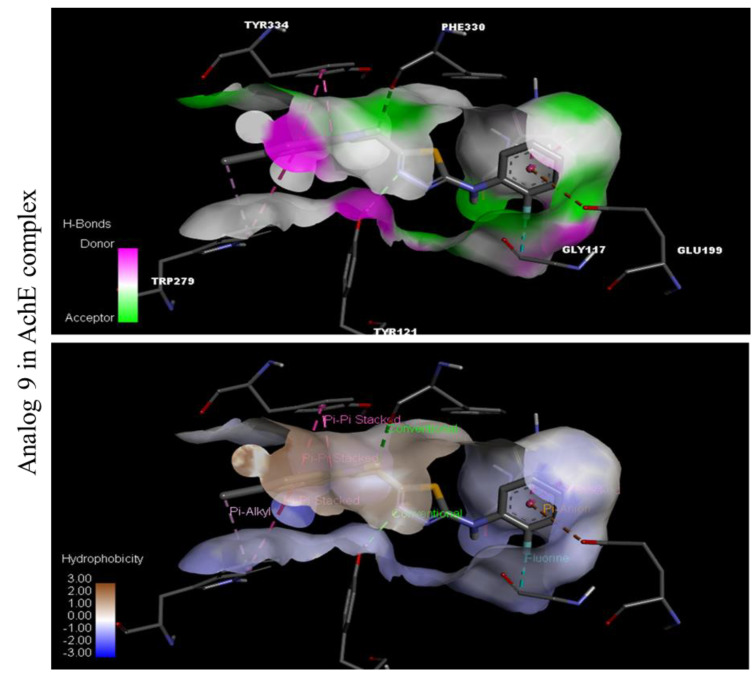
Indication of the hydrogen bond and hydrophobic nature against AChE of compound **9**.

**Figure 7:**
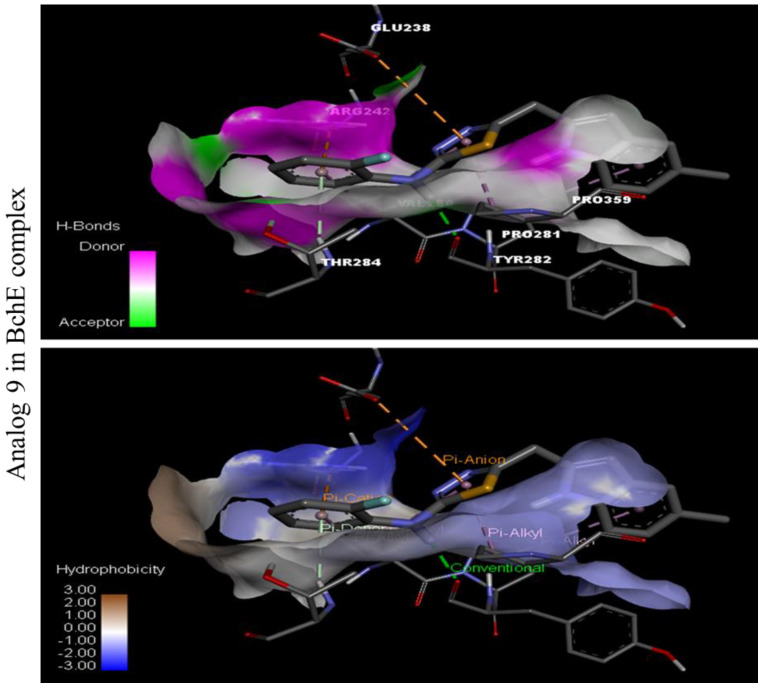
Indication of the hydrogen bond and hydrophobic nature against BuchE of compound **9**.

Ligand and protein preparation steps were done through a stepwise mechanism. In the initial stage, the removing of water molecules and preserving the protein and ligand in PDB format, the recovered protein was initially opened in auto dock vina. By the addition of polar hydrogen and charges like kollman and gasteiger, the docking procedure was continued. After protein preparation, the ligand was introduced. Automatic charges were applied to the ligand molecule, and torsion tree selection was made to find the root. After generating the configuration and saving the X, Y, and Z axis files in PDBQT format, the configuration was finally created. In order to create the many ligands poses needed to study the binding interaction between the ligand and active sites due to the abundance of protein interaction sites, a command prompt was employed. Figure 2, Figure 3, Figure 4, Figure 5, Figure 6 and Figure 7 illustrate the analysis of the binding interaction of the ligand with the protein’s active sites using nine distinct poses that were gathered in PDBQT format and analyzed in DSV.

### Docking Results

To investigate the mechanism in which synthesized scaffolds attach to the specified enzymes, acetylcholinesterase (AChE) and butyrylcholinesterase (BuChE), molecular docking was carried out. From www.rcsb.org, (28 January 2022) both enzyme crystallographic coordinates were obtained. When tested against the targeted enzymes, the docking strategy shows that the chosen compounds had a wide range of potentials. Among the examined series, several substituted scaffolds interact with various amino acids, but in this case, scaffolds **8** and **9** were found to have great interactions because they both had a flouro-group in the para- or ortho-position of an aromatic ring. Higher hydrogen bond counts result in greater interactions with enzyme active sites. As a result, the flouro-substituted scaffolds 8 and 9 were discovered to have strong interactions, as illustrated in Figure 2, Figure 3, Figure 4, Figure 5, Figure 6 and Figure 7 with a superposed surface complex.

In particular, the protein–ligand interaction (PLI) profile in the case of effective compounds 8 and 9 not only had the highest potential (in silico), but also displayed greater potency during an in vitro investigation. The potential of the molecule was found to be better as compared to the standard drug Donepezil, perhaps due to the presence of a flouro-group on the aromatic ring. This flouro-group is involved in making a hydrogen bond, which reduces the enzyme potential.

Figure 2 describes the protein ligand interaction profile (PLI) for compound 8 with flouro-groups in para-position revealed a variety of interacting residues for AChE, including GLU-199 (Hydrogen bonding), TRP-84 (Hydrogen bonding), TYR-121 (Hydrogen bonding), TYR-334 (Hydrogen bonding), PHE-330 (Hydrogen bonding) and GLU-199 (π-anion). As illustrated in Figure 2, the interacting residues for BuChE include TYR-282 (T-shaped), GLU-238 (H-F), VAL-280 (π-R), ARG-242 (π-cation), ASN-241 (Hydrogen bond with flourine), VAL-279 (Hydrogen bond with flourine), and PRO-281 (π-Alkyl).

As a result of different PLI profiles, Scaffold 9 exhibited better interactions. As illustrated in Figure 5, this molecule, which has a flouro-moiety at the aromatic ring, also contains the interacting residues against AchE that were TRP-279 (π-π stacked), PHE-330 (Hydrogen bonding), TYR-121 (Hydrogen bonding), GLY-117 (Hydrogen bond with flourine), GLU-199 (π-anion), TRP-84 (π-π-stacked), and TYR-334 (π-π-stacked). In contrast, as demonstrated in Figure 5, against BuChE, they were ARG-242 (π-anion), VAL-280 (π-R), PRO-359 (π-R), GLU-238 (π-cation), PRO-281 (π-Alkyl), and TYR-282 (Hydrogen bonding), among others.

The attached substituents and PLI profiles are the only differences between compounds 8 and 9. Both cases showed that the indole and thiadiazole moieties had a strong interface with the enzyme active site. Additionally, the nucleophilic nature of aromatic rings is enhanced by flouro-groups linked to the para and ortho sites.

## 5. Conclusions

Overall studies of the present work were achieved against AChE and BuChE, which represent scaffolds **8** (0.15 ± 0.050 and 0.20 ± 0.10 μM, respectively) and **9** (0.35 ± 0.050 and 0.50 ± 0.050 μM, respectively), which were found to be most potent among the tested series when compared to Donepezil, which was used as the standard drug (IC_50_ = 0.21 ± 0.12 and 0.30 ± 0.32 μM, respectively). Due to strong hydrogen interactions with enzyme active sites, the flouro-group at para- and ortho-positions on the aromatic ring clearly displays the inhibitory profiles of scaffolds. The interactions were further confirmed through molecular docking studies in which both scaffolds were identified through a varied range of interactions and their PLI profile was much better than other substituent compounds. Hydrogen bonds, pi–pi interactions, pi–sulfur interactions, and pi–cation interactions were employed to study how the scaffolds interacted. Due to attached rings and their heteroatoms, the scaffolds demonstrated all the specified interactions. Other substituted analogues were also found with good or moderate activity, which might be due to the presence of different groups among the series, as some compounds possess bulky groups like bromine, by which activity profiles were reduced and compounds were considered as poor inhibitors due to a much lower number of interactions with enzymes active sites.

## Figures and Tables

**Table 1:** Indole-based thiadiazole (**1–16**) derivatives and their acetylcholinesterase and butyrylcholinesterase inhibition profile.

S. No.	R	AChE IC_50_ (µM ± SEM)	BuChE IC_50_ (µM ± SEM)
**1**	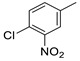	2.10 ± 0.10	2.80 ± 0.20
**2**	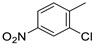	1.90 ± 0.10	4.30 ± 0.10
**3**	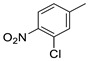	2.90 ± 0.10	4.60 ± 0.10
**4**	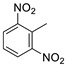	2.80 ± 0.10	5.40 ± 0.10
**5**	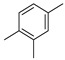	5.80 ± 0.20	9.30 ± 0.10
**6**	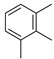	8.70 ± 0.20	10.90 ± 0.10
**7**	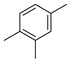	9.60 ± 0.20	13.60 ± 0.30
**8**	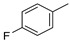	0.15 ± 0.010	0.20 ± 0.10
**9**	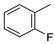	0.35 ± 0.050	0.50 ± 0.050
**10**	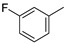	1.10 ± 0.10	2.70 ± 0.10
**11**	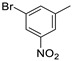	12.30 ± 0.20	18.40 ± 0.30
**12**	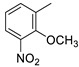	14.70 ± 0.30	19.20 ± 0.30
**13**	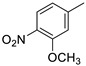	19.10 ± 0.30	25.30 ± 0.40
**14**	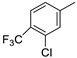	0.40 ± 0.050	2.60 ± 0.10
**15**	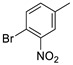	22.20 ± 0.40	32.10 ± 0.10
**16**	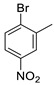	36.160 ± 0.50	34.30 ± 0.60
Standard Donepezil		0.21 ± 0.12	0.30 ± 0.32

## Data Availability

The datasets generated during and/or analyzed during the current study are available from the corresponding author upon reasonable request.

## Supplementary Materials

The following supporting information can be downloaded at: https://www.mdpi.com/article/10.3390/molecules27217368/s1, Figure S1: HNMR spectra of compound-1; Figure S2: HNMR spectra of compound-11; Figure S3: HNMR spectra of compound-16 [35,36,37,38].

## Sample Availability

Not available.
