# Supplementary material for: Synthesis, In Vitro Biological Evaluation and In Silico Molecular Docking Studies of Indole Based Thiadiazole Derivatives as Dual Inhibitor of Acetylcholinesterase and Butyrylchloinesterase
Source: Molecules. 2022 Oct 29;27(21):7368. doi: 10.3390/molecules27217368 (PMC9657098; doi:10.3390/molecules27217368)
Supplement: Supplementary file 1 [file molecules-27-07368-s001.zip › molecules-1954830-supplementary.pdf]

Synthesis, In Vitro Biological Evaluation and In Silico Molecular Docking Studies of Indole Based Thiadiazole Derivatives as Dual Inhibitor of Acetylcholinesterase and Butyrylchloinesterase

Shoaib Khan[1]*, Shahid Iqbal[2]*, Muhammad Taha[3], Fazal Rahim[1], Mazloom Shah[4], Hayat Ullah[5], Ali Bahadur[6]*, Hamad Alrbyawi[7], Ayed A. Dera[8], Mohammed Issa Alahmdi[9], Rami Adel Pashameah[10], Eman Alzahrani[11], Abd-ElAziem Farouk[12]

[1]*Department of Chemistry, Hazara University, Mansehra-21120, Pakistan.*

[2]*Department of Chemistry, School of Natural Sciences (SNS), National University of Science and Technology (NUST), H-12, Islamabad, 46000, Pakistan.*

[3]*Department of Clinical Pharmacy, Institute for Research and Medical Consultations (IRMC), Imam Abdulrahman Bin Faisal University, P.O. Box 31441, Dammam, Saudi Arabia*

[4]*Department of Chemistry, Abbottabad University of Science and Technology (AUST) Abbottabad, Pakistan.*

[5]*Department of Chemistry, University of Okara, Okara-56300, Punjab, Pakistan.*

[6]*Department of Chemistry, College of Science and Technology Wenzhou-Kean University, Wenzhou, China.*

[7]*Department of Chemistry, COMSATS University Islamabad campus-45550, Islamabad, Pakistan.*

[8]*Pharmaceutics and Pharmaceutical Technology Department, College of Pharmacy, Taibah University, Medina 42353, Saudi Arabia.*

[9]*Department of Clinical Laboratory Sciences, College of Applied Medical Sciences, King Khalid University, Abha, Saudi Arabia.*

[10]*Department of Chemistry, Faculty of Science, University of Tabuk, Tabuk- 71491, Saudi Arabia.*

[11]*Department of Chemistry, Faculty of Applied Science, Umm Al-Qura University, Makkah 24230, Saudi Arabia.*

[12]*Department of Chemistry, College of Science, Taif University, P.O. Box 11099, Taif 21944, Saudi Arabia.*

***To whom corresponding should be addressed**

shahidgcs10@yahoo.com (Shahid Iqbal), shoaibkhanswati@gmail.com (Shoaib Khan) and abahadur@wku.edu.cn (Ali Bahadur)

2.0 **Experimental**

*2.1 **Material and methods***

*2.1.1 General procedure of indole-based thiadiazole derivatives (**1-16**)*

All the required chemicals and their reagent were purchased from Sigma Aldrich USA and purity of solvent were obtained by distillations processes. Reaction was carried out in a fume hood and proper investigations were maintained by checking the reaction progress after every 30 minute. Different substituted isothiocyanate and hydrazine hydrate were mixed in THF and refluxed the reaction mixture for about 4hrs to obtain thiosemicarbazide derivatives which were treated with indole molecule having an aldehyde moiety in the presence of acetic acid, under refluxed condition for about 5hrs, afforded Schiff bases as an intermediate (**III**). These intermediates were cyclized after 16hrs refluxed in 1,4-dioxane in the presence of I_2/K_2CO_3, obtained indole based thiadiazole derivatives (**1-16**). The completion of reaction was confirmed by monitored TLC. All synthesized compounds were recrystallized in methanol. The purity of compounds was confirmed by proton NMR as well as HR-MS spectroscopic technique.

2.2 ***Spectral analysis***

2.2.1. 5-((5-chloro-1H-indol-2-yl)methyl)-N-(4-chloro-3-nitrophenyl)-1,3,4-thiadiazol-2-amine (1)

Yield: (78%); ^{1}HNMR (600 MHz, DMSO-d_6): δ 11.84 (s, 1H, NH), 11.32 (s, 1H, NH), 8.45 (d, 1H, J = 1.9 Hz, Indole-H), 8.33 (s, 1H, Ar-H), 8.26 (d, 1H, J = 7.3Hz, Ar-H), 8.14 (d, 1H, J = 7.9 Hz, Ar-H), 7.43 (d, 1H, J = 8.1 Hz, Indole-H), 6.51 (d, 1H, J = 7.4 Hz, Indole-H), 6.39 (s, 1H, Indole-H), 3.55 (s, 2H, CH_2); ^{13}CNMR (150 MHz, DMSO-d_6) δ 148.3, 147.2, 135.0, 132.5, 129.1, 126.3, 123.2, 123.3, 121.4, 121.0, 120.0, 119.6, 118.7, 118.1, 116.5, 115.5, 48.1; HREI-MS: m/z 419.9071; $[M+1]^+$ Calcd for $C_{17}H_{11}Cl_2N_5O_2S$; 419.8997.

2.2.2. 5-((5-chloro-1H-indol-2-yl)methyl)-N-(2-chloro-4-nitrophenyl)-1,3,4-thiadiazol-2-amine (2)

Yield: (74%); ^{1}HNMR (600 MHz, DMSO-d_6): δ 11.90 (s, 1H, NH), 11.40 (s, 1H, NH), 8.50 (d, 1H, J = 1.7 Hz, Indole-H), 836 (s, 1H, Ar-H), 830 (d, 1H, J = 7.3 Hz, Ar-H), 8.19 (d, 1H, J = 8.0 Hz, Ar-H), 7.90- 7.50 (d, 1H, J = 7.8 Hz, Indole-H), 6.57 (d, 1H, J = 7.4 Hz, Indole-H), 6.45 (s, 1H, Indole-H), 3.60 (s, 2H, CH_2); ^{13}CNMR (150 MHz, DMSO-d_6) δ 148.7, 147.3, 135.3, 132.1, 129.4, 126.7, 123.6, 123.0, 121.8, 121.2, 121.0, 120.0, 119.8, 118.3, 116.4, 115.7, 48.5; HREI-MS: m/z 419.9973; $[M+1]^+$ Calcd for $C_{17}H_{12}Cl_2N_4S$; 419.9997.

2.2.3. 5-((5-chloro-1H-indol-2-yl)methyl)-N-(3-chloro-4-nitrophenyl)-1,3,4-thiadiazol-2-amine (3)

Yield: (76%); ^{1}HNMR (600 MHz, DMSO-*d*$_6$): δ 11.88 (s, 1H, NH), 11.38 (s, 1H, NH), 8.48 (d, 1H, *J* = 1.7 Hz, Indole-H), 828 (d, 1H, *J* = 8.3 Hz, Ar-H), 8.17 (d, 1H, *J* = 7.7 Hz, Ar-H), 8.12 (s, 1H, Ar-H), 7.84 (t, *J* = 7.8 Hz, 1H, Ar-H), 7.69 (s, 1H, Ar-H), 7.48 (d, 1H, *J* = 7.1 Hz, Indole-H), 6.55 (d, 1H, *J* = 7.9 Hz, Indole-H), 6.42 (s, 1H, Indole-H), 3.57 (s, 2H, CH_2); ^{13}CNMR (150 MHz, DMSO-d$_6$) δ 148.5, 147.0, 135.1, 132.7, 129.2, 126.6, 123.3, 123.4, 121.5, 121.1, 121.0, 120.6, 119.7, 118.1, 116.0, 115.6, 48.3; HREI-MS: m/z 419.9969; $[M+1]^+$ Calcd for $C_{17}H_{11}Cl_2N_5O_2S$; 419.9997.

2.2.4. 5-((5-chloro-1H-indol-2-yl)methyl)-N-(2,6-dinitrophenyl)-1,3,4-thiadiazol-2-amine (4)

Yield: (69%); ^{1}HNMR (600 MHz, DMSO-*d*$_6$): δ 12.05 (s, 1H, NH), 11.92 (s, 1H, NH), 8.80 (d, 1H, *J* = 2.1 Hz, Indole-H), 8.54 (dd, 1H, *J* = 8.0, 1.9 Hz, Ar-H), 8.26 (dd, 1H, *J* = 8.1, 2.3 Hz, Ar-H), 7.77 (t, 1H, *J* = 7.9 Hz, Ar-H), 7.59 (d, 1H, *J* = 6.8 Hz, Indole-H), 6.62 (d, 1H, *J* = 7.2 Hz, Indole-H), 6.58 (s, 1H, Indole-H), 3.70 (s, 2H, CH_2); ^{13}CNMR (150 MHz, DMSO-d$_6$) δ 149.7, 148.3, 136.3, 133.1, 130.4, 127.7, 124.0, 123.4, 122.8, 122.2, 122.0, 121.0, 120.8, 119.3, 118.7, 117.4, 49.5; HREI-MS: m/z 430.0275; $[M+1]^+$ Calcd for $C_{17}H_{11}ClN_6O_4S$; 430.0292.

2.2.5. 5-((5-chloro-1H-indol-2-yl)methyl)-N-(3,4-dimethylphenyl)-1,3,4-thiadiazol-2-amine (5)

Yield: (65%); ^{1}HNMR (600 MHz, DMSO-*d*$_6$): δ 11.76 (s, 1H, NH), 11.23 (s, 1H, NH), 8.38 (d, 1H, *J* = 1.8 Hz, Indole-H), 8.19 (d, 1H, *J* = 7.1Hz, Ar-H), 8.07 (d, 1H, *J* = 7.6 Hz, Ar-H), 7.82 (s, 1H, Ar-H),7.38 (d, 1H, *J* = 8.0 Hz, Indole-H), 6.45 (d, 1H, *J* = 7.3 Hz, Indole-H), 6.30 (s, 1H, Indole-H), 3.45 (s, 2H, CH_2), 2.23 (s, 6H, CH3); ^{13}CNMR (150 MHz, DMSO-d$_6$) δ 147.3, 146.2, 134.0, 131.5, 128.1, 125.3, 122.4, 122.1, 120.4, 120.0, 119.0, 118.6, 117.7, 117.1, 115.5, 115.3, 47.1, 20.8, 20.2; HREI-MS: m/z 368.0524; $[M+1]^+$ Calcd for $C_{19}H_{17}ClN_4S$; 368.0543.

2.2.6. 5-((5-chloro-1H-indol-2-yl)methyl)-N-(2,3-dimethylphenyl)-1,3,4-thiadiazol-2-amine (6)

Yield: (71%); ^{1}HNMR (600 MHz, DMSO-*d*$_6$): δ 11.80 (s, 1H, NH), 11.26 (s, 1H, NH), 8.39 (d, 1H, *J* = 1.9 Hz, Indole-H), 8.24 (dd, 1H, *J* = 7.1, 2.0 Hz, Ar-H), 8.12 (dd, 1H, *J* = 7.0, 2.4 Hz, Ar-H), 7.89 (t, 1H, *J* = 6.9Hz, Ar-H), 7.41 (d, 1H, *J* = 8.0 Hz, Indole-H), 6.50 (d, 1H, *J* = 7.3 Hz, Indole-H), 6.35 (s, 1H, Indole-H), 3.51 (s, 2H, CH_2), 2.32 (s, 6H, CH3); ^{13}CNMR (150 MHz, DMSO-d$_6$) δ 147.9, 146.3, 134.8, 131.4, 128.0, 125.2, 122.3, 122.0, 120.3, 120.7, 119.2, 118.4, 117.6, 117.3, 115.8, 115.2, 47.0, 20.9, 20.4; HREI-MS: m/z 368.0527; $[M+1]^+$ Calcd for $C_{19}H_{17}ClN_4S$; 368.0543.

2.2.7. 5-((5-chloro-1H-indol-2-yl)methyl)-N-(3,4-dimethylphenyl)-1,3,4-thiadiazol-2-amine (7)

Yield: (76%); ^{1}HNMR (600 MHz, DMSO-*d*$_6$): δ 11.81 (s, 1H, NH), 11.28 (s, 1H, NH), 8.41 (d, 1H, *J* = 1.6 Hz, Indole-H), 8.26 (d, 1H, *J* = 7.7 Hz, Ar-H), 8.14 (d, 1H, *J* = 8.0 Hz, Ar-H), 7.84 (s, 1H, Ar-H), 7.43 (d, 1H, *J* = 7.0 Hz, Indole-H), 6.52 (d, 1H, *J* = 8.3 Hz, Indole-H), 6.38 (s, 1H, Indole-H), 3.53 (s, 2H, CH_2), 2.36 (s, 6H, CH3); ^{13}CNMR (150 MHz, DMSO-d$_6$) δ 147.7, 146.6, 134.5, 131.9, 128.4, 125.6, 122.2, 122.4, 121.3, 120.3, 119.8, 118.6, 117.7, 117.0, 115.5, 115.3, 47.5, 21.0, 20.8; HREI-MS: m/z 368.0521; $[M+1]^+$ Calcd for $C_{19}H_{17}ClN_4S$; 368.00543.

2.2.8. 5-((5-chloro-1H-indol-2-yl)methyl)-N-(4-fluorophenyl)-1,3,4-thiadiazol-2-amine (8)

Yield: (65%); ^{1}HNMR (600 MHz, DMSO-*d*$_6$): δ 11.60 (s, 1H, NH), 11.15 (s, 1H, NH), 8.20 (d, 1H, *J* = 1.8 Hz, Indole-H), 8.07 (d, 2H, *J* = 7.5Hz, Ar-H), 7.75 (d, 2H, *J* = 7.2 Hz, Ar-H), 7.25 (d, 1H, *J* = 8.4 Hz, Indole-H), 6.52 (d, 1H, *J* = 7.7 Hz, Indole-H), 6.44 (s, 1H, Indole-H), 3.40 (s, 2H, CH_2); ^{13}CNMR (150 MHz, DMSO-d$_6$) δ 149.3, 147.2, 136.0, 133.5, 130.1, 128.3, 124.4, 123.1, 122.4, 121.0, 120.0, 119.6, 118.7, 117.8, 116.5, 115.3, 49.5; HREI-MS: m/z 358.0277; $[M+1]^+$ Calcd for $C_{17}H_{12}ClFN_4S$; 358.0292.

2.2.9. 5-((5-chloro-1H-indol-2-yl)methyl)-N-(2-fluorophenyl)-1,3,4-thiadiazol-2-amine (9)

Yield: 80%); ^{1}HNMR (600 MHz, DMSO-*d*$_6$): δ 11.59 (s, 1H, NH), 11.20 (s, 1H, NH), 8.15 (d, 1H, *J* = 1.8 Hz, Indole-H), 8.04 (dd, 1H, *J* = 7.5, 2.3 Hz, Ar-H), 7.78 (dd, 1H, *J* = 7.2, 1.9 Hz, Ar-H), 7.65-7.64 (m, 1H, Ar-H), 7.45-7.41 (m, 1H, Ar-H), 7.20 (d, 1H, *J* = 8.3 Hz, Indole-H), 6.45 (d, 1H, *J* = 6.7 Hz, Indole-H), 6.47 (s, 1H, Indole-H), 3.20 (s, 2H, CH_2); ^{13}CNMR (150 MHz, DMSO-d$_6$) δ 150.6, 149.3, 148.2, 139.0, 135.5, 132.1, 130.3, 128.4, 125.1, 124.4, 123.0, 122.0, 121.6, 120.7, 119.8, 118.5, 45.5; HREI-MS: m/z 358.0275; $[M+1]^+$ Calcd for $C_{17}H_{12}ClFN_4S$; 358.0292.

2.2.10. 5-((5-chloro-1H-indol-2-yl)methyl)-N-(3-fluorophenyl)-1,3,4-thiadiazol-2-amine (10)

Yield: (77%); ^{1}HNMR (600 MHz, DMSO-*d*$_6$): δ 11.57 (s, 1H, NH), 11.24 (s, 1H, NH), 8.17 (d, 1H, *J* = 1.8 Hz, Indole-H), 8.06 (dd, 1H, *J* = 7.4, 2.2 Hz, Ar-H), 7.79 (dd, 1H, *J* = 7.0, 1.8 Hz, Ar-H), 7.63 (t, *J* = 8.0Hz, 1H, Ar-H), 7.43 (s, 1H, Ar-H), 7.21 (d, 1H, *J* = 7.3 Hz, Indole-H), 6.46 (d, 1H, *J* = 6.9 Hz, Indole-H), 6.48 (s, 1H, Indole-H), 3.25 (s, 2H, CH_2); ^{13}CNMR (150 MHz, DMSO-d$_6$) δ 150.6, 149.6, 148.4, 139.3, 135.9, 132.5, 130.6, 128.1, 125.3, 124.2, 123.9, 122.8, 121.7, 120.9, 119.7, 118.0, 45.6; HREI-MS: m/z 358.0274; $[M+1]^+$ Calcd for $C_{17}H_{12}ClFN_4S$; 358.0292.

2.2.11. N-(3-bromo-5-nitrophenyl)-5-((5-chloro-1H-indol-2-yl)methyl)-1,3,4-thiadiazol-2-amine (11)

Yield: (68%); ^{1}HNMR (600 MHz, DMSO-d_6): δ 11.86 (s, 1H, NH), 11.50 (s, 1H, NH), 8.77 (d, 1H, J = 2.1 Hz, Ar-H), 8.49 (d, 1H, J = 2.0 Hz, Ar-H), 8.33 (s, 1H, Ar-H), 7.55 (d, 1H, J = 8.5 Hz, Indole-H), 6.77 (d, 1H, J = 8.3 Hz, Indole-H), 6.59 (d, 1H, J = 1.8 Hz, Indole-H), 6.53 (s, 1H, Indole-H), 2.97 (s, 2H, CH_2); ^{13}CNMR (150 MHz, DMSO-d6) δ 151.5, 149.5, 148.2, 128.3, 127.7, 126.3, 125.7, 124.7, 122.9, 122.7, 121.8, 121.6, 118.6, 117.4, 111.8, 100.8, 48.75; HREI-MS: m/z 462.0472; $[M+1]^+$ Calcd for $C_{17}H_{11}ClBrN_5O_2S$; 462.0492.

2.2.12. 5-((5-chloro-1H-indol-2-yl)methyl)-N-(2-methoxy-3-nitrophenyl)-1,3,4-thiadiazol-2-amine (12)

Yield: (72%); ^{1}HNMR (600 MHz, DMSO-d_6): 11.76 (s, 1H, NH), 11.34 (s, 1H, NH), 8.37 (d, 1H, J = 2.4 Hz, Indole-H), 8.34 (dd, 1H, J = 7.1, 2.2 Hz, Ar-H), 8.27 (dd, 1H, J = 7.7, 2.0 Hz, Ar-H), 7.95 (t, 1H, J = 7.0 Hz, Ar-H, 7.40 (d, 1H, J = 8.3 Hz, Indole-H), 6.56 (d, 1H, J = 7.4 Hz, Indole-H), 6.29 (s, 1H, Indole-H), 3.68 (s, 2H, CH_2), 2.15 (s, 3H, -OCH3); ^{13}CNMR (150 MHz, DMSO-d_6) δ 148.6, 147.7, 135.4, 133.8, 130.3, 129.5, 128.1, 126.3, 125.2, 124.2, 120.7, 119.5, 118.6, 117.9, 113.4, 112.2, 46.4, 19.5; HREI-MS: m/z 415.0472; $[M+1]^+$ Calcd for $C_{18}H_{14}ClN_5O_3S$; 415.0492.

2.2.13. 5-((5-chloro-1H-indol-2-yl)methyl)-N-(3-methoxy-4-nitrophenyl)-1,3,4-thiadiazol-2-amine (13)

Yield: (75%); ^{1}HNMR (600 MHz, DMSO-d_6): δ 11.74 (s, 1H, NH), 11.33 (s, 1H, NH), 8.36 (d, 1H, J = 1.9 Hz, Indole-H), 8.33 (d, 1H, J = 8.1 Hz, Ar-H), 8.26 (d, 1H, J = 7.0 Hz, Ar-H), 7.91 (s, 1H, Ar-H) 7.45 (d, 1H, J = 7.6 Hz, Indole-H), 6.55 (d, 1H, J = 7.3 Hz, Indole-H), 6.27 (s, 1H, Indole-H), 3.66 (s, 2H, CH_2), 2.10 (s, 3H, -OCH3); ^{13}CNMR (150 MHz, DMSO-d_6) δ 148.7, 147.6, 135.5, 133.9, 130.4, 129.6, 128.2, 126.4, 125.3, 124.3, 120.8, 119.6, 118.7, 116.0, 113.5, 112.3, 46.5, 19.0; HREI-MS: m/z 415.0472; $[M+1]^+$ Calcd for $C_{18}H_{14}ClN_5O_3S$; 415.0492.

2.2.14. 5-((5-chloro-1H-indol-2-yl)methyl)-N-(3-chloro-4-(trifluoromethyl)phenyl)-1,3,4-thiadiazol-2-amine (14)

Yield: (68%); ^{1}HNMR (600 MHz, DMSO-d_6): δ 11.63 (s, 1H, NH), 11.59 (s, 1H, NH),), 8.27 (d, 1H, J = 2.8 Hz, Indole-H), 8.14 (d, 1H, J = 7.3 Hz, Ar-H), 7.73 (d, 1H, J = 7.1 Hz, Ar-H), 7.60 (s, 1H, Ar-H), 7.20 (d, 1H, J = 8.0 Hz, Indole-H), 6.36 (d, 1H, J = 7.6 Hz, Indole-H), 6.26 (s, 1H, Indole-H), 3.15 (s, 2H, CH_2); ^{13}CNMR (150 MHz, DMSO-d_6) δ 150.6, 150.3, 149.2, 146.0, 144.5, 139.1, 136.3, 134.4, 131.1, 130.4, 128.0, 125.0, 124.6, 123.7, 121.8, 120.5, 119.3, 53.5; HREI-MS: m/z 442.0239; $[M+1]^+$ Calcd for $C_{18}H_{11}Cl_2F_3N_4S$; 442.0260.

2.2.15. N-(4-bromo-3-nitrophenyl)-5-((5-chloro-1H-indol-2-yl)methyl)-1,3,4-thiadiazol-2-amine (15)

Yield: (73%); ^{1}HNMR (600 MHz, DMSO-d_6): δ 11.86 (s, 1H, NH), 11.39 (s, 1H, NH), 8.47 (d, 1H, J = 2.5 Hz, Indole-H), 8.39 (s, 1H, Ar-H), 8.29 (d, 1H, J = 7.3Hz, Ar-H), 8.16 (d, 1H, J = 7.5 Hz, Ar-H), 7.45 (d, 1H, J = 7.1 Hz, Indole-H), 6.53 (d, 1H, J = 8.4 Hz, Indole-H), 6.49 (s, 1H, Indole-H), 3.77 (s, 2H, CH_2); ^{13}CNMR (150 MHz, DMSO-d_6) δ 149.3, 148.2, 143.0, 142.5, 136.1, 133.3, 131.2, 128.3, 125.4, 124.0, 122.0, 121.6, 119.7, 118.1, 117.5, 116.5, 49.1; HREI-MS: m/z 462.2476; $[M+1]^+$ Calcd for $C_{17}H_{11}BrClN_5O_2S$; 462.2291.

2.2.16. N-(2-bromo-5-nitrophenyl)-5-((5-chloro-1H-indol-2-yl)methyl)-1,3,4-thiadiazol-2-amine (16)

Yield: (76%); ^{1}HNMR (600 MHz, DMSO-d_6): δ 11.88 (s, 1H, NH), 11.67 (s, 1H, NH), 8.78 (d, 1H, J = 7.0 Hz, Ar-H), 8.51 (d, 1H, J = 7.1 Hz, Ar-H), 8.42 (s, 1H, Ar-H), 7.67 (s, 1H, Indole-H), 7.58 (d, 1H, J = 2.9 Hz, Indole-H), 7.03 (d, 1H, J = 8.2 Hz, Indole-H), 6.60 (d, 1H, J = 7.2Hz, Indole-H), 3.81 (s, 2H, CH_2);^{13}CNMR (150 MHz, DMSO-d_6) δ 152.5, 150.5, 149.2, 129.3, 128.7, 127.3, 126.7, 125.7, 123.9, 123.7, 122.8, 122.6, 119.6, 118.4, 112.8, 101.8, 49.75; HREI-MS: m/z 462.0215; $[M+1]^+$ Calcd for $C_{17}H_{11}BrClN_5O_2S$; 462.0237.

The following spectra of the given compounds are shown in **figure S 1-3**.

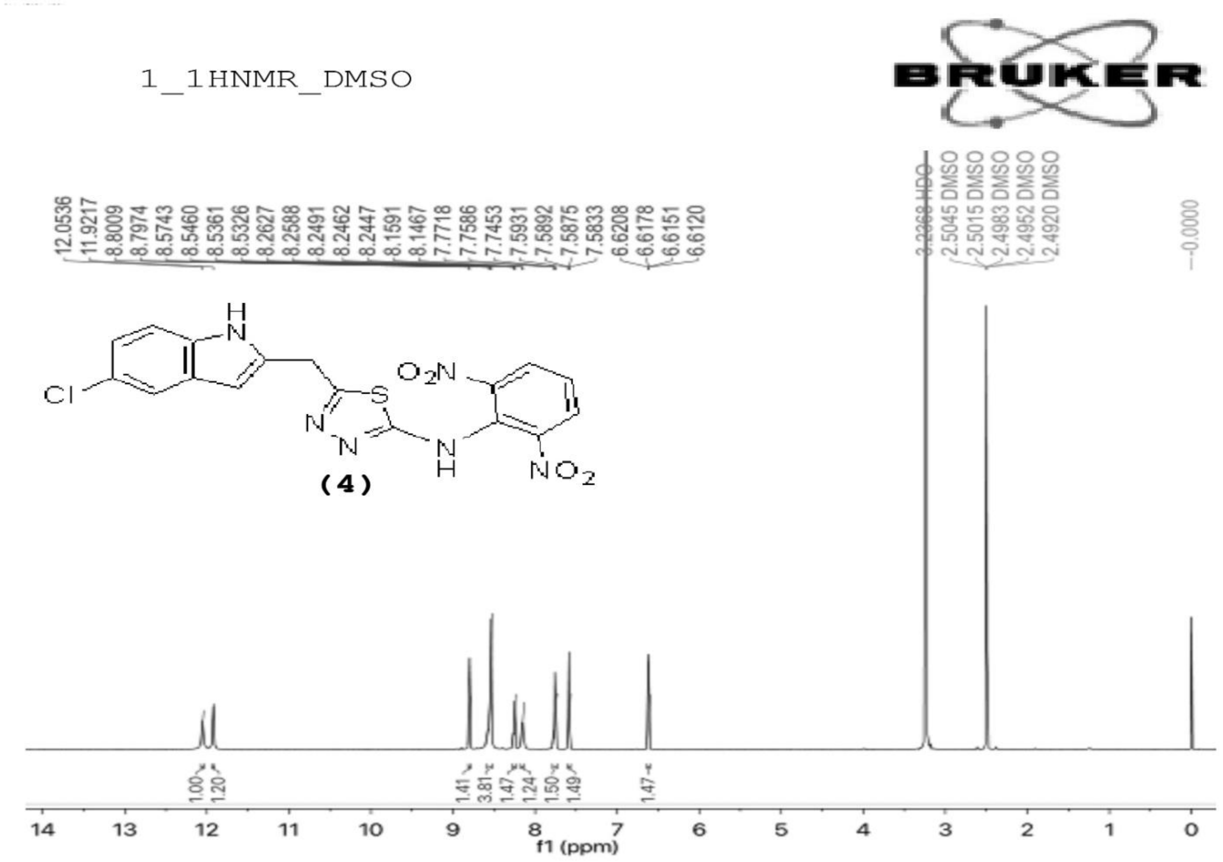

Figure S1 HNMR spectra of compound-1

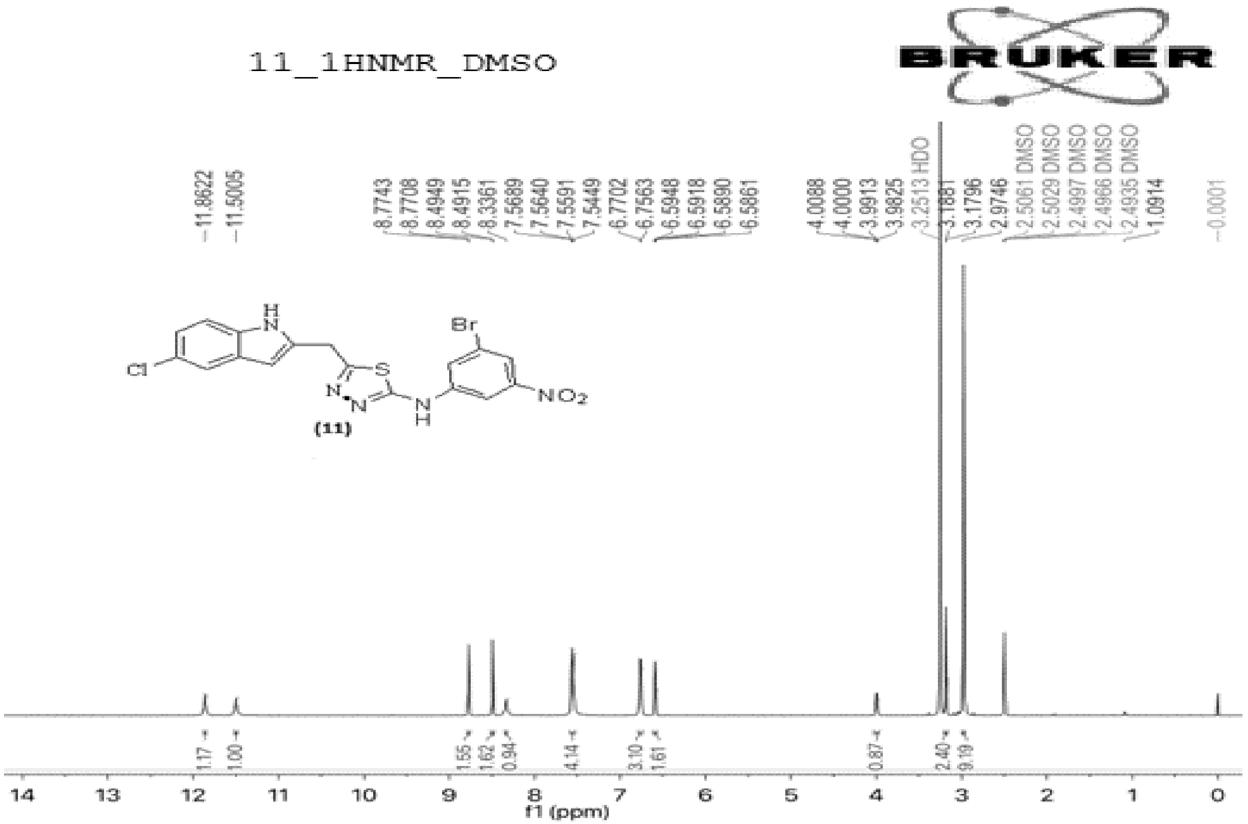

Figure S2 HNMR spectra of compound-11

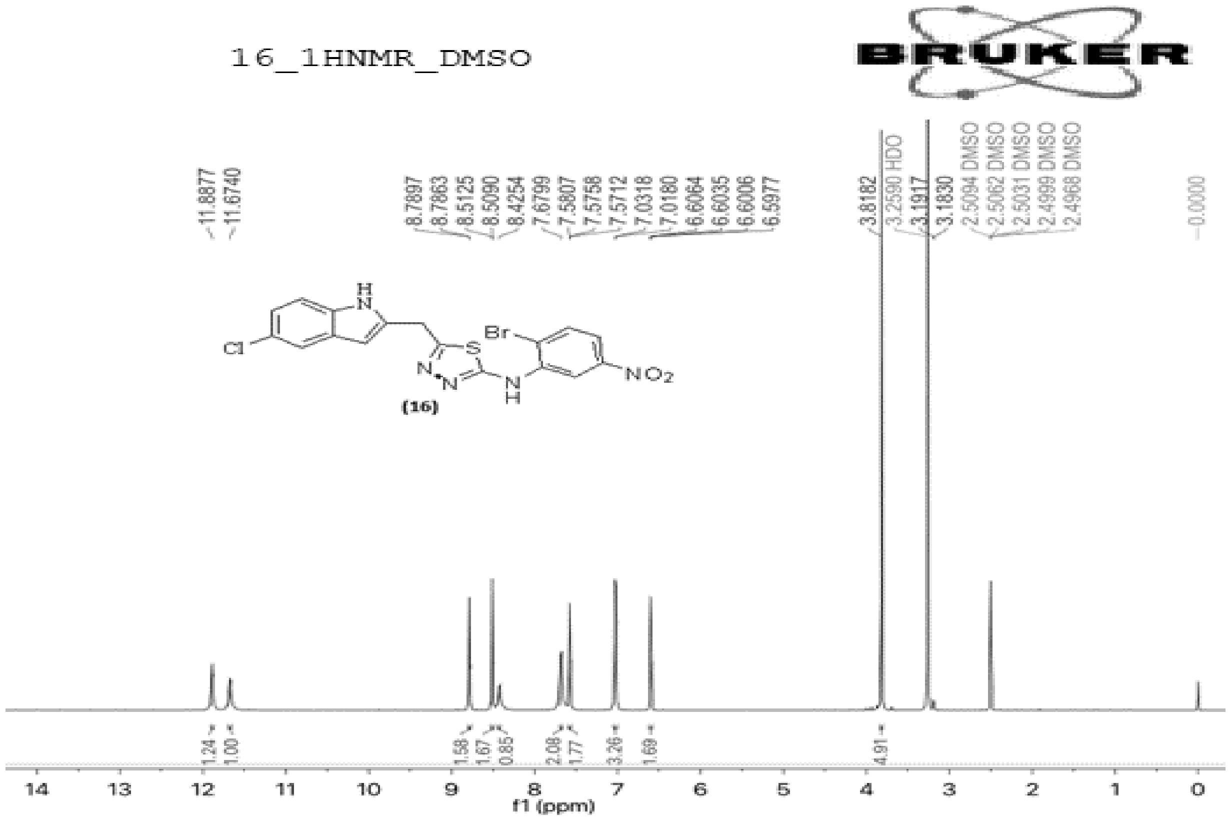

Figure S3 HNMR spectra of compound-16

3.4. Molecular docking protocol

According to Crippen's fragmentation method, the partition coefficient (log P) values were predicted using Crippen's fragmentation by the CS ChemProp module of ChemDraw Ultra 12 (Cambridge Soft, Cambridge, MA, USA). [35]. The polar surface area (tPSA) was calculated by the atom-based method [36]. Chemdraw (14.0) was used to generate the structures of compounds 8 and 9, which were then imported into the auto dock tool. Following a final check of all atom and bond types and the addition of any required hydrogen atoms, the Gasteiger-Marsili charges were assigned. Marvin determined the substances' pKa values and the ionisation states that correspond to physiological conditions (pH 7.4). (ChemAxon). The molecules were docked to alpha glucosidase and alpha amylase. It was necessary to build the enzyme structure before docking with GoldSuite (CCDC). The hydrogen atoms were added, certain water molecules (616, 634, and 643) were kept, and all of the histidine residues were protonated at Nε. All amino acid residues within a radius of around 12 from the known compound, acarbose, were considered to be within the binding site. There were used a common set of genetic algorithms. The

population size was 100, and there were 100,000 operations. Nine conformations of each chemical were as a result obtained. DSV provided results visualization (2020).

3.5. Acetylcholinesterase Activity Assay Protocol

The inhibition of AChE and BChE was determined using a method described earlier [37,38]. Briefly, the stock solutions (1mg/mL) of test analogues were prepared using DMSO. The working solutions (1–100µg/mL) were prepared using serial dilutions (a serial dilution means a series of diluted solutions, e.g., 0.1mg/mL, 0.2mg/mL and so on; the solutions contained 5% DMSO and 95% water). The various concentrations of test compounds (10µL) were pre-incubated with sodium phosphate buffer (0.1 M; pH 8.0; 150µL) and AChE (0.1 U/mL; 20µL) for 15 min at 25°C. The reaction was initiated via the addition of DTNB (1 mM; 10µL) and AChEI (1 mM; 10 µL). The mixture of reaction was mixed using a cyclomixer and incubated for 10 min at 25oC. The absorbance was measured using a microplate reader at a 410 nm wavelength against the blank reading containing 10µL DMSO instead of the test compound (the solution contained 5% DMSO and 95% water). Utilizing the formula given in Equation (1), the percentage of inhibition was computed, and the IC50 was determined under the positive control of Donepezil (0.01-100 g/mL).

% Inhibition = (Absorbance of control—Absorbance of compound)/Absorbance of control × 100

IC50 is the concentration of a drug or inhibitor required to inhibit 50% of an enzyme's activity which was calculated by constructing a non-linear regression graph between the percentages of inhibition vs. concentration, using Graph Pad prism software (version 5.3)(accessed on 5 August 2022).

References

35. Ghose, A.K.; Crippen, G.M. Atomic physicochemical parameters for three-dimensional structure-directed quantitative structure-activity relationships. 2. Modeling dispersive and hydrophobic interactions. *J. Chem. Inf. Comput. Sci.* **1987**, *27*, 21–35.
36. Ertl, P.; Rohde, B.; Selzer, P. Fast calculation of molecular polar surface area as a sum of fragment-based contributions and its application to the prediction of drug transport properties. *J. Med. Chem.* **2000**, *43*, 3714–3717.
37. Chigurupati, S.; Selvaraj, M.; Mani, V.; Selvarajan, K.K.; Mohammad, J.I.; Kaveti, B.; Bera, H.; Palaniuthu, V.R.; Teh, L.K.; Salleh, M.Z. Identification of novel acetylcholinesterase inhibitors: Indolopyrazoline derivatives and molecular docking studies. *Bioorganic Chem*. **2016**, *67*, 9–17.
38. Chigurupati, S.; Selvaraj, M.; Mani, V.; Mohammad, J.I.; Selvarajan, K.K.; Akhtar, S.S.; Marikannan, M.; Raj, S.; Teh,L. K.; Salleh, M.Z. Syn thesis of azomethines derived from cinnamal dehyde and vanillin: In vitro acetylcholinesterase inhibitory, antioxidant and in silico molecular docking studies. *Med. Chem. Res.* **2018**, *27*, 807—816.
